# The DUNDRUM-1 structured professional judgment for triage to appropriate levels of therapeutic security: retrospective-cohort validation study

**DOI:** 10.1186/1471-244X-11-43

**Published:** 2011-03-16

**Authors:** Grainne Flynn, Conor O'Neill, Clare McInerney, Harry G Kennedy

**Affiliations:** 1 National Forensic Mental Health Service, Central Mental Hospital, Dundrum, Dublin 14, Ireland; 2 Department of Psychiatry, Trinity College, Dublin, Ireland

## Abstract

**Background:**

The assessment of those presenting to prison in-reach and court diversion services and those referred for admission to mental health services is a triage decision, allocating the patient to the appropriate level of therapeutic security. This is a critical clinical decision. We set out to improve on unstructured clinical judgement. We collated qualitative information and devised an 11 item structured professional judgment instrument for this purpose then tested for validity.

**Methods:**

All those assessed following screening over a three month period at a busy remand committals prison (n = 246) were rated in a retrospective cohort design blind to outcome. Similarly, all those admitted to a mental health service from the same prison in-reach service over an overlapping two year period were rated blind to outcome (n = 100).

**Results:**

The 11 item scale had good internal consistency (Cronbach's alpha = 0.95) and inter-rater reliability. The scale score did not correlate with the HCR-20 'historical' score. For the three month sample, the receiver operating characteristic area under the curve (AUC) for those admitted to hospital was 0.893 (95% confidence interval 0.843 to 0.943). For the two year sample, AUC distinguished at each level between those admitted to open wards, low secure units or a medium/high secure service. Open wards v low secure units AUC = 0.805 (95% CI 0.680 to 0.930); low secure v medium/high secure AUC = 0.866, (95% CI 0.784 to 0.949). Item to outcome correlations were significant for all 11 items.

**Conclusions:**

The DUNDRUM-1 triage security scale and its items performed to criterion levels when tested against the real world outcome. This instrument can be used to ensure consistency in decision making when deciding who to admit to secure forensic hospitals. It can also be used to benchmark admission thresholds between services and jurisdictions. In this study we found some divergence between assessed need and actual placement. This provides fertile ground for future research as well as practical assistance in assessing unmet need, auditing case mix and planning care pathways.

## Background

The clinical assessment of patients referred for admission to therapeutically secure and other hospitals has seldom been studied. The systematic allocation of patients to appropriate levels of therapeutic security is however central to the operation of mental health services generally and especially forensic mental health services. This is an area of clinical decision making that is critical for the timely delivery of services to those who are severely mentally ill in prison or less secure hospitals. We set out to improve on unstructured professional judgement and existing instruments for assessment of need for therapeutic security. Our purpose is to provide a validated and reliable way of arriving at such decisions in a transparent way. The structured professional judgement approach would also lend itself to benchmarking and quality standards.

There is a literature concerning the assessment of need for therapeutic security in which cross sectional samples of those already in various levels of therapeutic security are compared. The earliest studies used unstructured professional judgment. One such study used a panel of research clinicians to examine the 'ideal' placement for prisoners identified in a survey as having a severe mental Illness [1]. Others followed this pattern, typically asking an 'expert' panel of researchers or clinicians to emulate real world decisions concerning the ideal placement for a subject based on standardised summaries of history and assessments of dependency needs [2-5].

Learmont [6] applied the facet method for sociological research [7] to describe an algorithm for allocating prisoners to levels of prison security according to whether they were dangerous to the public, an escape risk or had access to outside resources and help. This is of limited relevance when assessing for hospital admission.

Eastman & Bellamy's [8] Admission Criteria for Secure Services Schedule (ACSeSS) is a set of criteria used in needs assessment which could be read as a structured professional judgment instrument. This identified seven domains relevant to need for placement in secure settings including the gravity of recent or past violent behaviour, the immediacy of any risk of violent behaviour in the community or in hospital, psychopathology that 'predicts' the above, specialised psychopathology that specifically determines anti-social behaviour - specialist forensic need; the likely duration of the admission, unpredictability and lastly how the case would be perceived by a criminal justice agency - a 'trump' factor that might determine admission to a higher level of security than other factors would indicate. There are no published validation studies for these criteria and the assessment of 'likely duration' is unclear. Other approaches have included Coid & Kahtan's [9] algorithm based on severity of offence and legal category which is specific to one jurisdiction; Shaw et al's [10] structured professional judgment instrument was based on patient centered factors such as security needs, dependency needs, treatment needs, 'political' considerations and likely length of hospital stay using visual analogue ratings all rated using untethered Likert scales.

Kennedy [11] compiled definitions for various levels of therapeutic security based on institutional characteristics but also provided clinical criteria based on patient characteristics for the allocation or stratification of patients to these various levels of therapeutic security. The same paper gave suggested criteria for the movement of patients down through the levels of therapeutic security, or along a pathway towards recovery. This formed the starting point for the drafting of this set of structured professional judgment instruments.

Sugarman & Walker [12] adapted the HONOS, adding 'SECURE' items made up of severity items and physical, staffing and procedural items, mixing patient centred and institutional characteristics. Collins & Davies' [13] provided security centered factors such as physical security, relational security and procedural security. These have the advantage of detailed item definitions but emphasise institutional characteristics over patient centred features. The last two of these have in common a rating system designed to match patients to levels of security, from 0 to 4. An actuarial tool based on risk factors which contained only one item reflecting seriousness of violence had a moderate receiver operating characteristic but modest predictive power [14,15].

We have collated all of these themes combined with a review of existing custom and practice to devise a manual [16] consisting of four scales or sets of items relevant to (1) the decision to allocate a patient to a particular level of therapeutic security, (2) the urgency of that patient's need and therefore their place on the waiting list relative to others, (3) completion of treatment programmes aimed at reducing risk of violence and (4) the extent to which a patient shows signs of recovery or reduced need for therapeutic security. The first of these scales, the DUNDRUM-1 triage security instrument is investigated in this paper. It defines eleven items relevant to the decision to allocate a person to high or medium security, low security, open hospital beds or community follow-up. Each item is rated from 0 to 4 with examples given for what would constitute an appropriate rating. For each item, those rated '4' would appropriately be allocated to high security, those rated '3' to medium security, '2' to a low-secure unit (e.g. psychiatric intensive care or a locked high observation unit), '1' could be safely cared for in an open psychiatric ward and '0' could be followed up as an out-patient.

Our hypotheses were that the eleven items taken as a scale would have acceptable psychometric properties and the total score would distinguish between those admitted to different levels of therapeutic security while each of the 11 items should also correlate with outcome. We hypothesised also that the DUNDRUM-1 triage security scale would correlate weakly or not at all with the HCR-20, a measure of risk which is not designed to take account of the seriousness of the risk or complexity of treatment need.

## Methods

The study was approved by the local research ethics, audit and effectiveness committee. All data was stored in anonymised form.

### Study Design

This is a retrospective cohort study [17], instigated as part of the clinical audit and service evaluation process at the National Forensic Mental Health Service for Ireland. All those committed to a large remand (pre-trial) prison (Cloverhill Prison, Dublin) were screened by nurses and a general practitioner and those identified as possible cases were referred for full psychiatric assessment by a psychiatric prison in-reach and court diversion scheme. Cases were ascertained by an administrator from a case register of all those screened and assessed at Cloverhill prison in the relevant period. The register also included the eventual outcome of the contact.

The clinical notes and assessments of all those assessed by the psychiatric prison in-reach and court diversion scheme over a three month period April to June 2009 were rated by two senior clinicians (GF and CO'N) blind to the eventual outcome. A further overlapping sample identified in the same way (January 2008 to December 2009) consisted of all those who were diverted from the same remand prison to hospitals at various levels of therapeutic security over a two year period.

### Setting

Ireland has a population of 4.4 m and in December 2009 had a prison population of 4,200 including 660 who were remanded in custody pending trial. Cloverhill prison is the largest remand prison in the state, serving 70% of the population.

In the two years January 2008 to December 2009 7,454 men newly committed to Cloverhill prison were screened by nurses at the point of reception using a four item screening questionnaire [18]. 1,454 were identified for full psychiatric assessment by the psychiatric in-reach and court liaison service. Of these 100 were diverted from prison to psychiatric hospitals, including 27 sent to open wards in 16 local hospitals and 26 to low secure units (psychiatric intensive care or high dependency units) in three hospitals. These local hospitals are 'approved' to detain patients under the civil mental health act for Ireland, but are not 'designated' to detain patients under the criminal law insanity act for Ireland. A further 47 were diverted to the Central Mental Hospital, the sole forensic hospital for Ireland and the only hospital designated to receive patients detained under the Criminal Law (Insanity) Act 2006 for Ireland. This act permits the transfer from prison to the Central Mental Hospital of those remanded in custody or sentenced by the courts, if medically certified as having a mental disorder and in need of hospital treatment, as well as those found unfit to stand trial or not guilty by reason of insanity [19]. The Central Mental Hospital provides admission wards at medium and high secure levels [20]. The 100 admitted to hospital were assessed using the DUNDRUM-1 security triage scale [16]. The assessments were carried out as consensus ratings by the first two authors who were blind to the eventual outcomes.

A related sub-sample was further analysed, consisting of 921 new committals (receptions) screened between April and June 2009, of whom 246 were identified for full assessment by the psychiatric in-reach team and 30 were diverted from the criminal justice system to any hospital placement. The 246 were rated using the DUNDRUM-1 security triage scale [16] by the same two clinicians in the same way. The 30 diverted from the criminal justice system to hospital overlap with the 100 described in the previous paragraph. A total of 316 were fully assessed and rated with the DUNDRUM-1.

### Rating Scale: DUNDRUM-1: Triage Security Structured Assessment

The structured professional judgment instrument the DUNDRUM-1 is the product of an iterative drafting process. This commenced in early 2008 with a brainstorming and consultative session amongst the consultant forensic psychiatrists who are responsible for the decision to admit patients to the Central Mental Hospital. Nine consultant forensic psychiatrists were consulted, all of whom had worked or were working at the Central Mental Hospital. The nine had worked variously in nine medium or high secure forensic mental health services in five different jurisdictions. Colleagues in other disciplines were also consulted. The second phase consisted of an iterative process of refinement of definitions based on observation of discussions and practice at the weekly referrals meeting at the Central Mental Hospital at which all referrals are discussed and assessments prioritised. This meeting is chaired by the consultant forensic psychiatrist on call for that week and is attended by the leaders of all multi-disciplinary teams (consultant forensic psychiatrists), the heads of all disciplines (nursing, psychology, social work, occupational therapy), nurses in charge of wards and hospital managers. Clinicians from the psychiatric court liaison and prison in-reach service in the main remand prison also attend and those providing in-reach clinics in the other prisons. Referrals for assessment with a view to admission from local mental health units are allocated to consultant forensic psychiatrists and when assessed these are also considered for admission at this meeting. The structured professional judgment instrument described here - the DUNDRUM-1 triage security instrument is part of the 22^nd ^revision of this draft. It forms part of a suite of structured professional judgment instruments [16] along with the DUNDRUM-2, an instrument for assessing the urgency of need for admission and prioritisation of waiting lists, and two instruments for assessing readiness for movement to less secure places, the DUNDRUM-3 programme completion instrument and the DUNDRUM-4 recovery instrument.

**Figure 1:**
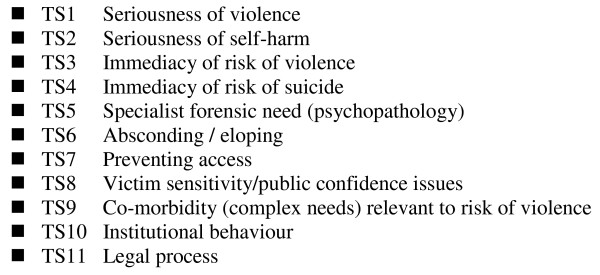
**DUNDRUM-1 Triage Security Items**. For full content, see [16]

The assessment of the appropriate level of therapeutic security for those requiring mental health interventions was assessed using an 11 item scale (figure 1 and additional file 1). Each item is rated using a five point scale from 0 (no security needed, or no mental disorder), 1 (could be managed in an open hospital ward), 2 (could be managed in a local psychiatric intensive care ward/low secure unit), 3 (could be managed in a medium secure unit) and 4 (special/high security required). The ratings for each item are tethered to operational definitions [16] (and additional file 1).

Ratings were made jointly by two senior clinicians based on the unstructured but detailed pre-admission assessments and court reports prepared by the psychiatric in-reach team. These ratings were prepared blind to the eventual outcome of the case. There was no missing data. This reflects the relevance of the item content.

For cross-validation, the HCR-20 [21] 'H' and 'C' items were assessed on a sub-sample. The clinicians making the ratings were trained in the use of the HCR-20 and were blind to outcome.

### Outcome Measure

For the three month diversion sample, the eventual disposal of each case was rated on a three point scale as (1) discharged to the general practitioner in the prison, (2) followed up by the psychiatric in-reach team in the prison or community mental health team, and (3) admitted to any of the available hospitals.

For the two year sample of all those transferred from the remand prison to hospital, the outcome was rated on a three point scale as admitted to an open hospital ward, admitted to a local psychiatric intensive care unit (locked low secure unit) or admitted to the Central Mental Hospital, where in practice the admission ward does not distinguish between medium or high security.

For item-outcome correlations in the combined sample, outcome was measured on a four point scale (0) not admitted, (1) admitted to an open ward, (2) admitted to a PICU and (3) admitted to the Central Mental Hospital (combined medium and high security).

### Statistics

All data were entered for analysis in SPSS-16 [22]. All data were stored anonymously.

Inter-rater reliability was calculated by arranging for a rater (CO'N) to rate a consecutive series of cases (n = 18) from a two week period, blind to the ratings of GF. Cohen's kappa and Spearman's rank correlation test were calculated for items and the total scores were also correlated.

Factor analysis was carried out using principle components analysis without rotation. Internal consistency was assessed using Cronbach's Alpha, examining whether the item to total correlations were improved by the omission of individual items.

To calculate receiver operating characteristics, outcomes were dichotomized as above or below a given level of therapeutic security.

For item to outcome correlations, Spearman rank correlations were used. For item to outcome correspondence, for each item at each score the observed proportion actually allocated to the corresponding or higher level of therapeutic security was regarded as a positive while those allocated to a lower level of therapeutic security were rated nil, and the binomial probability was calculated for the Z-approximation based on a test proportion of 0.5 (random correspondence). As a more rigorous test, the proportions allocated to the level of therapeutic security exactly corresponding with the rating were also examined.

## Results

### Inter-rater reliability

Two clinicians rated 18 cases independently and blind to each other. The Kappa statistic could be calculated for 7 of the 11 items and was greater than 0.85 for each (p < 0.001 for each). For all 11 items, Spearman's rank correlation coefficient was greater than 0.75, (p < 0.001). The total score for the eleven items correlated 0.959 (p < 0.001).

### Internal Consistency

Ratings were available for 316 people assessed using the DUNDRUM-1 security triage scale [16]. Exploratory factor analysis yielded a first factor with Eigen value 7.48 which accounted for 67.9% of the variance. This loaded positively on all nine items other than the two items concerning self harm or suicide. The second factor had an Eigen value of 1.5 and accounted for 13.7% of the variance and loaded positively only on the two self harm/suicide items.

A measure of internal consistency, Cronbach's Alpha was 0.949 for the eleven-item scale. Table 1 shows that the corrected item-total correlation was greater than 0.8 for 9 of the eleven items. Only the two items relating to self harm had low item-total correlations but removing either of these items increased the Alpha statistic only from 0.949 to 0.957.

**Table 1:** Internal consistency

	Corrected Item-Total Correlation	Cronbach's Alpha if Item Deleted
TS1: serious violence	.833	.942

TS2: serious self harm	.352	.956

TS3: immediacy of violence risk	.882	.940

TS4: immediacy of self harm risk	.295	.957

TS5: specialist forensic need	.887	.941

TS6: absconding risk	.936	.938

TS7: preventing access	.893	.939

TS8: victim sensitivities	.822	.943

TS9: complex risks	.839	.941

TS10: institutional behaviour	.803	.944

TS11: legal procedure	.898	.941

Cronbach's Alpha statistic for DUNDRUM-1 triage security scale (11 items), n = 316. Overall Alpha = 0.949.

### Cross-Validation

The HCR-20 [21] ratings for historical and clinical (current) items were available for 32 individuals. The HCR-20 historical items correlated with the DUNDRUM-1 security triage scale using Spearman rank correlation coefficient r = 0.329 (NS) and the HCR-20 current items correlated r = 0.166 (NS).

### Triage for Court Diversion

For the three month period April to June 2009 table 2 shows that of 246 persons assessed 159 were discharged to the prison GP for follow up, 57 were followed in the psychiatric in-reach clinic and 30 were admitted to a psychiatric hospital. The total score on the DUNDRUM-1 triage security scale differed significantly for the three groups (ANOVA F = 360.1, df = 2, p < 0.001). The 95% confidence intervals did not overlap and post-hoc tests using Bonferroni's method showed that each of the three groups differed significantly from the other two.

**Table 2:** Total DUNDRUM-1 score by outcome for those assessed following screening over a three month period

				95% Confidence interval	
	**N**	**Mean**	**SD**		

Discharge to GP	159	0.21	0.79	0.09	0.34

Psychiatric follow up	57	4.14	4.57	2.93	5.35

Admit to psychiatric hospital	30	15.77	5.33	13.78	17.76

Total	246	3.02	5.82	2.29	3.75

DUNDRUM-1 triage security score (sum of 11 items) for 246 male remand prisoners assessed following screening April to June 2009, by outcome.

The receiver operating characteristic for the threshold between discharge to the GP (n = 159) and either psychiatric follow-up or admission (n = 87) yields an area under the curve (AUC) = 0.893 (95% confidence interval 0.843 to 0.943) with sensitivity at a score of 1 = 0.782 and specificity = 0.922.

**Figure 2:**
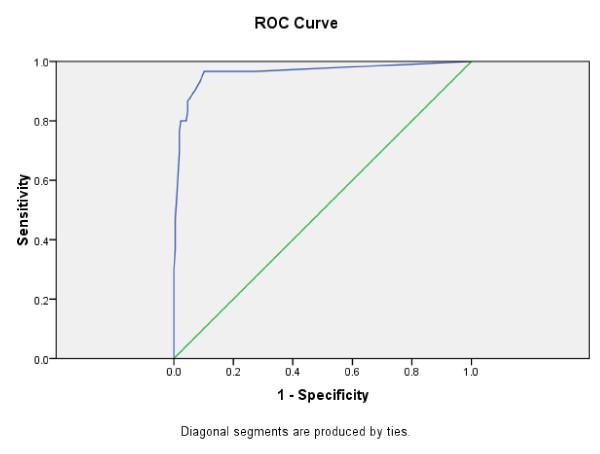
**DUNDRUM-1 Triage Security score**. April to June 2009, those not transferred from prison to hospital (n = 216) v transferred from prison to any hospital (n = 30) Area Under the Curve = 0.984 (95% confidence interval 0.971 to 0.997)

The receiver operating characteristic for the distinction between those admitted to any psychiatric hospital during this three month period (n = 30) and those not admitted (n = 216) yields area under the curve = 0.984 (95% confidence interval 0.971 to 0.977) (figure 2). At a cut off score of 6, sensitivity was 0.95 and specificity 0.92.

### Triage for Various Levels of Therapeutic Security

Table 3 shows that for the two year period January 2008 to December 2009 100 individuals were either diverted from the remand prison to local psychiatric hospitals in open wards or locked low secure intensive care units (PICUs), or transferred from the prison to the forensic hospital, the Central Mental Hospital at medium/high security. The total scores on the DUNDRUM-1 triage security 11-item scale differed significantly according to the level of security to which the person was admitted (ANOVA F = 75.2, df = 2, p < 0.001) and as before each group differed significantly from the other two as assessed by the Bonferroni test for post hoc differences.

**Table 3:** Total score by outcome for those admitted to hospital following screening over a two year period

				95% Confidence interval	
	**N**	**Mean**	**SD**	**Lower**	**Upper**

Open wards	27	10.74	3.26	9.45	12.03

PICU	26	15.81	4.39	14.03	17.58

Medium and high secure	47	22.87	4.56	21.53	24.21

Total	100	17.76	6.64	16.44	19.07

DUNDRUM-1 triage security score (sum of 11 items) for 100 male remand prisoners admitted to hospital following screening and assessment over a two year period 2008-2009. ANOVA F = 75.2, df = 2, p < 0.001.

To calculate receiver operating characteristics, those admitted were divided into three groups and the area under the curve calculated for adjacent pairs of groups - open wards -v- psychiatric intensive care units, psychiatric intensive care units -v- medium/high security. These comparisons yield conservative estimates of the receiver operating characteristics. Larger areas under the curve are generated and better sensitivity and specificity calculated if all those above or below a level of security are compared.

**Figure 3:**
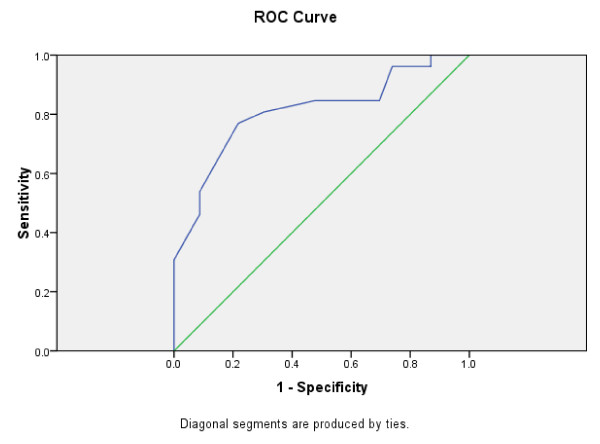
**DUNDRUM-1 Triage Security score**. Two year period 2008 to 2009, those transferred to open wards (n = 27) compared to those transferred to psychiatric intensive care units (n = 26) Area Under the Curve = 0.805 (95% confidence interval 0.680 to 0.930)

For those admitted to open wards (n = 27) compared to those admitted to psychiatric intensive care units (n = 26), the receiver operating characteristic yielded an area under the curve of 0.805 (95% confidence interval 0.680 to 0.930) (figure 3) and at a threshold score of 13 sensitivity was 0.78, specificity 0.71.

**Figure 4:**
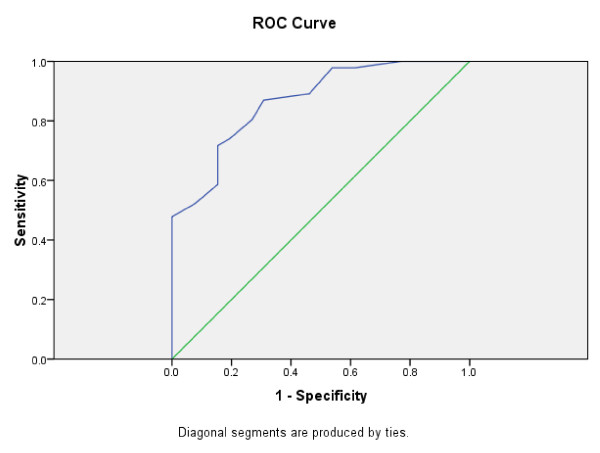
**DUNDRUM-1 Triage Security score**. Two year period 2008 to 2009, those transferred to psychiatric intensive care units (n = 26) compared to those transferred to forensic medium and high security at Central Mental Hospital (n = 47) Area Under the Curve = 0.866 (95% confidence interval 0.784 to 0.949)

For those admitted to psychiatric intensive care units (locked low secure units, n = 26) compared to those admitted to a forensic medium/high secure unit (n = 47) the receiver operating characteristic area under the curve was 0.866 (95% confidence interval 0.784 to 0.949) (figure 4) and at a threshold score of 20 sensitivity was 0.728, specificity 0.827.

Re-analysing the differences between levels of security for a nine item scale omitting the two items for seriousness of self harming history and immediacy of self-harm made no significant difference to any of these results.

### Items and Outcomes

Table 4 shows that the Spearman rank correlation between items and outcomes for nine violence related items ranged between 0.755 and 0.874. The two suicide-related items had the weakest item to outcome correlations at 0.270 and 0.248, though these were still statistically significant.

**Table 4:** Item to outcome correlations and exact agreement with outcome

	**Observed proportion in Agreement (n = 316)**^**a**^	95% CI of observed proportion in agreement	**Spearman rank correlation coefficient**^**b**^
TS1: serious violence	0.75	0.70 - 0.80	0.803

TS2: serious self harm	0.61	0.56 - 0.67	0.259

TS3: immediacy of violence risk	0.75	0.70 - 0.80	0.879

TS4: immediacy of self harm risk	0.67	0.62 - 0.73	0.236

TS5: specialist forensic need	0.78	0.74 - 0.83	0.908

TS6: absconding risk	0.80	0.76 - 0.85	0.879

TS7: preventing access	0.78	0.74 - 0.83	0.831

TS8: victim sensitivities	0.80	0.76 - 0.85	0.806

TS9: complex risks	0.72	0.67 - 0.77	0.828

TS10: institutional behaviour	0.71	0.66 - 0.76	0.758

TS11: legal procedure	0.92	0.90 - 0.95	0.921

Exact agreement i.e. those allocated to the level of therapeutic security corresponding to the rating - 0 not followed up or followed up as an out-patient, 1 admitted to open ward, 2 admitted to PICU, 3 or 4 admitted to forensic (medium or high secure) service. Spearman rank correlation coefficient for five point rating (0 to 4) against outcome in four levels. N = 316. ^a^Binomial test for observed proportions differing from test proportion 0.5, based on Z approximation all p < 0.001 (2-tailed), ^b ^all p < 0.001

The measurement of exact agreement between the rating and the level of therapeutic security to which an individual was allocated is also shown in table 4. The proportion of agreement in table 4 is the sum of those rated '0' who had a corresponding outcome 'not admitted', those rated '1' who were admitted to an open ward, those rated '2' who were admitted to a PICU and those rated '3' or '4' who were admitted to the forensic hospital (medium and high security). Agreement for items ranged between 61% and 92%. The two suicide related items had agreement with outcome of 61% and 67%, comparable to the agreement for the violence related items. The 'legal procedure' item had the strongest agreement with outcome at 92%, while several items related to patient focused issues and social context rated higher than simple violence related items, including absconding risk (80%), victim sensitivities (80%), preventing access to contraband (78%) and specialist forensic treatment needs (78%).

Table 5 shows the relationship between item scores and the level of therapeutic security to which the person was eventually allocated where agreement was rated if the allocation was at the corresponding level of therapeutic security or higher. Those allocated to a lower level of therapeutic security than the rating indicated were regarded as non-corresponding.

**Table 5:** Item to outcome agreement where outcome is matched to appropriate level or higher.

	All assessed over a three month period April to June 2009 (n = 246)		All diverted from prison to hospital over a two year period 2008-2009 (n = 100)	
	**Observed proportion in Agreement**^**a**^	**99% CI of observed proportion in agreement**	**Observed proportion in Agreement**^**a**^	**99% CI of observed proportion in agreement**

TS1: serious violence	.85	.79 - .91	.88	.79 - .97

TS2: serious self harm	.91	.86 - .96	.98	.94 - .99

TS3: immediacy of violence risk	.89	.84 - .94	.72	.60 - .84

TS4: immediacy of self harm risk	.97	.94 - .99	.99	.96 - .99

TS5: specialist forensic need	.96	.92 - .99	.95	.89 - .99

TS6: absconding risk	.89	.85 - .95	.92	.84 - .99

TS7: preventing access	.92	.87 - .96	.90	.82 - .98

TS8: victim sensitivities	.93	.89 - .97	.81	.71 - .91

TS9: complex risks	.81	.75 - .87	.79	.68 - .89

TS10: institutional behaviour	.91	.86 - .96	.99	.96 - .99

TS11: legal procedure	.95	.92 - .99	.90	.82 - .98

First sample all assessed after screening over a three month period, n = 246; second sample all diverted to hospitals over a two year period n = 100. ^a^Binomial test for observed proportions differing from test proportion 0.5, based on Z approximation all p < 0.001 (2-tailed).

Tables 6 and 7 show the relationship between the ratings and the actual placement for each item. Table 6 shows the percentages of agreement between each rating for each item and the actual placement, where the placement matches the rating or is at a higher (safer) level of therapeutic security. Table 7 shows the percentages of those placed at each level of therapeutic security who were rated on each item as appropriate for that placement or a less secure placement.

**Table 6:** Matching of rating for each item and eventual placement.

	RATING				
					**Total**

	**0**	**1**	**2**	**3-4**	**(n = 316)**

TS1: serious violence	188/188 (100%)	54/85 (65%)	12/15 (80%)	22/28 (79%)	276 (87%)

TS2: serious self harm	267/267 (100%)	12/34 (35%)	7/7 (100%)	6/8 (75%)	292 (92%)

TS3: immediacy of violence risk	199/199 (100%)	9/23 (39%)	47/75 (63%)	16/19 (84%)	271 (86%)

TS4: immediacy of self harm risk	294/294 (100%)	3/9 (33%)	8/10 (80%)	3/3 (100%)	308 (97%)

TS5: specialist forensic need	214/214 (100%)	47/54 (87%)	32/36 (89%)	11/12 (92%)	304 (96%)

TS6: absconding risk	197/197 (100%)	20/37 (54%)	50/62 (81%)	19/20 (95%)	286 (91%)

TS7: preventing access	211/211 (100%)	21/25 (84%)	39/59 (66%)	19/21 (90%)	290 (92%)

TS8: victim sensitivities	228/228 (100%)	15/18 (83%)	12/23 (52%)	32/47 (68%)	289 (91%)

TS9: complex risks	180/180 (100%)	32/63 (51%)	25/36 (69%)	22/37 (59%)	259 (82%)

TS10: institutional behaviour	208/208 (100%)	59/79 (75%)	20/23 (87%)	6/6 (100%)	293 (93%)

TS11: legal procedure	209/209 (100%)	17/20 (85%)	28/38 (74%)	44/49 (90%)	298 (94%)

WEIGHTED MEAN % TOTALS	2398/2398 (100%)	298/447 (65%)	283/381 (74%)	200/250 (80%)	3166/3476 (91%)

For those given each rating of each item (0 = community, 1 = open ward, 2 = PICU, 3 or 4 forensic medium or high secure), the number and percentage placed correctly or at a higher (safer) level of therapeutic security. Note that for those rated '0', any placement in the community or at a higher level is appropriate.

**Table 7:** Matching of eventual placements with ratings for each item

	PLACEMENT				
	**Community**	**Open ward**	**PICU**	**Medium or high security**	**Total**

	**N = 216**	**N = 27**	**N = 26**	**N = 47**	**(n = 316)**

TS1: serious violence	184 (85%)	24 (89%)	21 (81%)	47 (100%)	276 (87%)

TS2: serious self harm	194 (90%)	26 (96%)	25 (96%)	47 (100%)	292 (92%)

TS3: immediacy of violence risk	196 (91%)	5 (19%)	23 (88%)	47 (100%)	271 (86%)

TS4: immediacy of self harm risk	208 (96%)	27 (100%)	26 (100%)	47 (100%)	308 (97%)

TS5: specialist forensic need	208 (96%)	27 (100%)	25 (96%)	47 (100%)	307 (97%)

TS6: absconding risk	194 (90%)	19 (70%)	26 (100%)	47 (100%)	286 (91%)

TS7: preventing access	200 (93%)	19 (70%)	24 92%)	47 (100%)	290 (92%)

TS8: victim sensitivities	207 (96%)	19 (70%)	16 62%)	47 (100%)	289 (91%)

TS9: complex risks	180 (83%)	16 (59%)	16 (62%)	47 (100%)	259 (82%)

TS10: institutional behaviour	194 (90%)	26 (96%)	26 (100%)	47 (100%)	293 (93%)

TS11: legal procedure	207 (96%)	18 (67%)	26 (100%)	47 (100%)	298 (94%)

WEIGHTED MEAN % TOTALS	21732 (91%)	208 (68%)	254 (89%)	517 (100%)	3168 (91%)

For each placement, the number and percentage of those allocated to that level of therapeutic security who had ratings for each item indicating that the placement or a lower level of security was appropriate. Note that for those placed in medium or high security (n = 47), any rating of 4 or less is consistent with a safe placement (100%).

## Discussion

This is a retrospective-cohort study. Because a complete cohort of those screened forms the basis for the cohort studied and the ratings were made blind to outcome and based on information gathered prior to the outcome, this is a valid and cost-effective study design [17]. We found that the eleven DUNDRUM-1 triage security items perform well as a scale, with good inter-rater reliability and good internal consistency. The DUNDRUM-1 has good receiver operating characteristics distinguishing between the levels of therapeutic security to which mentally disordered remand prisoners were diverted. We have also demonstrated that each of the eleven items correlated with outcome. We believe this instrument has advantages over other instruments [8-10,12,13,15] because it assesses patient centred rather than institutional factors; because, with a related paper [Davoren et al, submitted] it has been validated according to the criteria recommended by the Risk Management Authority of Scotland [23] and because it is drafted in a form that is likely to be applicable across jurisdictions and services.

The scale does not correlate significantly with the HCR-20 'H' score, a measure of static risk of violence, nor does it correlate with the HCR-20 'C' score, an assessment of dynamic risk factors for violence. This indicates that in measuring the need for therapeutic security, the DUNDRUM-1 measures something other than the risk of violence. Because the DUNDRUM-1 has concomitant validity for the level of therapeutic security to which a patient is allocated, it would appear that it measures something considered in practice to be of greater importance than risk, at least for this decision. A prospective study to test the predictive validity of the HCR-20 in comparison with the DUNDRUM-1 would be needed to further clarify this.

### Patient and clinician factors

The statistical separation between scores at different levels of therapeutic security overall suggests some syndromal association of patterns or profiles of ratings, with those rated '0' on one item more likely to rate '0' on others, while the same tended to hold true for those rated '4'. This is in keeping with the factor analysis.

Ratings and outcomes diverge from complete agreement as revealed in table 4. This reflects the pragmatism and patient focus inherent in professional judgment. A high overall score should not bind the clinical decision maker. Equivalent scores might be generated by ten items all rated '2' and five items all rated '4'. But the former patient could be safely cared for in a PICU which the latter is likely to need admission to a forensic unit at medium or high secure level. In some cases a single '4' might justify a specialist forensic admission. Table 5 shows the pragmatic use of higher levels of security than apparently required, though there are also notable 'slippages' apparent in tables 6 when some were allocated to levels of therapeutic security below those assessed as needed, and table 7 when some of those allocated to low security (PICU) or open wards had ratings indicating that a higher level of security was more appropriate. This disparity was highest for open wards, perhaps reflecting their greater availability. For future research, this might be addressed by increased availability of PICU places.

### System and population factors

The data presented here reflect the dynamic interplay of population demand, the availability of alternative facilities in the community or at lower levels of therapeutic security [24] and resource allocation, so that actual admission thresholds arising from the balance of these factors are likely to vary from time to time and from one jurisdiction or administrative region to another. The DUNDRUM-1 triage security scale performs well in practice even in this naturalistic study of actual outcomes. Some of those admitted to PICUs may have had scores or clinical profiles more typical of those admitted to medium security and vice versa (see tables 6 and 7) - this is the reality of clinical practice. Even under these constraints the scores were sufficiently distinct to distinguish the actual allocation to levels of security as measured by the receiver operating characteristic. Although the AUC is a property of the instrument, not of the population tested, the threshold scores are a property of the population studied and so the threshold scores found in this study might not generalize to another jurisdiction, for a number of reasons. The most important is that the threshold for admission to any given level of security will fall when more beds are available and will rise when the number of beds is smaller. A complex relationship can also be expected between the availability of beds at one level of therapeutic security and the demand for beds at adjacent levels [24] and this can influence time spent on waiting lists [25]. We believe that because the threshold scores may vary between services and jurisdictions, the threshold scores can be used to make valid benchmark comparisons between services and jurisdictions.

### Triage and urgency of need for admission

We are conscious of the ethical and organisational aspects of triage admission decision making. This will be discussed in greater detail in a related article.

The items comprising the security triage scale are intended to be predominantly 'static' or 'fixed' in nature though some may be responsive to change over time to a limited extent. The urgency of need for treatment is a dynamic quality that can change from day to day or week to week. A separate rating scale, the DUNDRUM-2 urgency triage scale is currently being validated for this purpose [16].

Because the items comprising the DUNDRUM-1 security triage scale are static in nature, separate scales for recovery and treatment completion are also being validated as measures of the extent to which progress in treatment can offset the need for therapeutic security, leading to progress from high to low secure placements and eventually to follow-up in the community [16].

### Limitations

Although the researchers made ratings blind to outcomes, the same sources of information guided the actual decision makers, so there may be some degree of halo effect which could only be overcome by a true prospective study.

## Conclusions

The assessment of need for therapeutic security can be described for practical purposes by the eleven items described here. The eleven items covered may not be exhaustive, but they afford an assessment which appears to account for most of the variance in placements in this jurisdiction. We found that the scale has good receiver operating characteristics in this retrospective cohort study and each item correlated with outcome. Replication in other jurisdictions and other mental health systems is essential. We believe these items and this scale are sufficiently general to apply elsewhere.

This structured professional judgment instrument is different from risk assessment tools. This should not however be seen as the return of 'dangerousness'. It would be more accurate to see this as a particular assessment of specialist dependency needs.

We believe this instrument will have utility in auditing the appropriateness of placements at the decision making stage. However the ordering of waiting lists will depend on additional 'urgency' items to be described in a subsequent paper. Similarly, we doubt that this instrument would be helpful in assessing progress in treatment and decreasing need for therapeutic security - risk assessment instruments such as the HCR-20 are already validated for this function and have the advantage of including dynamic ratings, sensitive to change. This instrument should however be a means of matching experimental and control groups in studies of treatment outcome in forensic patient groups. We have also prepared structured professional judgment instruments that will reflect decreasing need for therapeutic security[16], along the lines suggested in Kennedy [11].

## Competing interests

The authors declare that they have no competing interests.

## Authors' contributions

GF collected and entered data and coordinated the project. GF and CO'N rated the data and contributed to the item definitions in the manual. CMcI coordinated the case register and outcome data. HGK produced the first draft of the manual, designed the study and carried out the data analysis. All contributed to the authorship of the paper.

## Pre-publication history

The pre-publication history for this paper can be accessed here:

http://www.biomedcentral.com/1471-244X/11/43/prepub

## Supplementary Material

Additional file 1 **The DUNDRUM-1 manual**. This additional file contains the full item definitions and rating scales for the eleven items of the DUNDRUM-1 triage security scale. See also [16]. Click here for file

## References

[B1] Gunn J Maden A Swinton M Treatment needs of prisoners with psychiatric disorders BMJ 1991 303 338 340 10.1136/bmj.303.6798.338 1912775 PMC1670792

[B2] Maden A Curle C Meaux C Burrow S Gunn J The treatment and security needs of patients in special hospitals Criminal Behaviour & Mental Health 1993 3 290 306 10.1002/cbm.503 12897900

[B3] Shaw J McKenna J Snowden P Boyd C McMahon D Kilshaw J The North West Region 1: clinical features and placement needs of patients detained in special hospitals J Forensic Psychiatry 1994 5 93 105 10.1080/09585189408410900

[B4] Pierzchniak P Farnham F de Taranto N Bull D Gill H Bester P McCallum A Kennedy H Assessing the needs of patients in secure settings. A multi-disciplinary approach Journal of Forensic Psychiatry 1999 10 343 354 10.1080/09585189908403688

[B5] O'Neill C Heffernan P Goggins R Corcoran C Linehan S Duffy D O'Neill H Smith C Kennedy HG Long-stay forensic psychiatric inpatients in the republic of Ireland: aggregated needs assessment Irish Journal of Psychological Medicine 2003 20 119 125 10.1017/S0790966700007916 30308720

[B6] Learmont J Review of Prison Service Security in England and Wales and the Escape from Parkhurst Prison on Tuesday 3rd January 1995 1995 London, HMSO Cm3020

[B7] Cantor D Facet Theory: Approaches to Social Research 1985 New York: Springer-Verlag

[B8] Eastman N Bellamy S Admission Criteria for Secure Services Schedule (ACSeSS) 1998 London, St Georges Hospital Medical School

[B9] Coid J Kahtan N An instrument to measure the security needs of patients in medium security Journal of Forensic Psychiatry 2000 11 119 134

[B10] Shaw J Davies J Morey H An Assessment of the security, dependency and treatment needs of all patients in secure services in a UK health region Journal of Forensic Psychiatry 2001 12 610 637 10.1080/09585180110092010

[B11] Kennedy HG Therapeutic Uses of Security: mapping forensic mental health services by stratifying risk Advances in Psychiatric Treatment 2002 8 433 443 10.1192/apt.8.6.433

[B12] Sugarman PA Walker L HoNOS-SECURE version 2 2004 London: Royal College of Psychiatrists College Research and Teaching Unit

[B13] Collins M Davies S The Security Needs Assessment Profile: a multi-dimensional approach to measuring security needs International Journal of Forensic Mental Health 2005 4 39 62

[B14] Brown CSH Lloyd K Comparing clinical risk assessments using operationalised criteria Acta Psychiatrica Scand 2002 supplement 142 148 151 10.1034/j.1600-0447.106.s412.32.x 12072148

[B15] Brown CSH Lloyd K OPRISK: a structured checklist assessing security needs for mentally disordered offenders referred to high security hospital Criminal Behaviour & Mental Health 2008 18 190 202 10.1002/cbm.689 18618502

[B16] Kennedy HG O'Neill C Flynn G Gill P The Dundrum Toolkit. Dangerousness, Understanding, Recovery and Urgency Manual (The Dundrum Quartet) V1.0.22 (18/03/10). Four Structured Professional Judgment Instruments for Admission Triage, Urgency, Treatment Completion and Recovery Assessments 2010 Dublin, Ireland, Trinity College Dublin http://hdl.handle.net/2262/39131

[B17] Zahner GEP Hsieh CC Fleming JA Tsuang MT, Tohen M, Zahner GEP Introduction to epidemiological research methods Textbook in Psychiatric Epidemiology 1995 New York, Wiley-Liss pp36 37

[B18] Birmingham L Gray J Mason D Grubin D Mental illness at reception into prison Criminal Behaviour and Mental Health 2000 10 77 87 10.1002/cbm.347

[B19] Kennedy H The Annotated Mental Health Acts 2007 Dublin, Blackhall Publishing

[B20] Pillay SM Oliver B Butler L Kennedy HG Risk stratification and the care pathway Irish Journal of Psychological Medicine 2008 25 123 127 10.1017/S0790966700011228 30282248

[B21] Webster CS Douglas KS Eaves D Hart SD HCR-20: Assessing risk for violence, version 2 1997 Burnaby, British Columbia: Simon Fraser University

[B22] SPSS 16 for Windows Release 16.0.1 November 15, 2007

[B23] Risk Management Authority of Scotland Risk Assessment Tools Evaluation Directory (RATED) version 1 2006 Scotland: Astron http://www.rmascotland.gov.uk

[B24] O'Grady J The complimentary role of regional and local secure provision for psychiatric patients Health Trends 1990 22 14 16 10105448

[B25] Pierzchniak P Purchase N Kennedy HG Liaison between Court, Prison and Psychiatric Services Health Trends 1997 29 26 29

