# Supplementary material for: The DUNDRUM-1 structured professional judgment for triage to appropriate levels of therapeutic security: retrospective-cohort validation study
Source: BMC Psychiatry. 2011 Mar 16;11:43. doi: 10.1186/1471-244X-11-43 (PMC3066108; doi:10.1186/1471-244X-11-43)
Supplement: Additional file 1 — The DUNDRUM-1 manual. This additional file contains the full item definitions and rating scales for the eleven items of the DUNDRUM-1 triage security scale. See also [16]. [file 1471-244X-11-43-S1.DOC]

**DUNDRUM-1: TRIAGE SECURITY ITEMS**

The triage items should be distinguished qualitatively from the items included in structured professional judgment tools for risk assessment such as the HCR-20. The Triage items are divided here into DUNDRUM-1 Triage Security items and DUNDRUM-2 Triage Urgency items.

The triage items are all predicated on there being an established mental disorder present, whether mental illness, mental impairment or dementia, or any other legal category in the jurisdiction in which the instrument is to be used. In accordance with international conventions such as COE Rec(10)2004, intoxication and social deviance are excluded from mental disorder. It is made clear in the definitions that absence of a mental disorder leads to a ‘zero’ rating. Diagnosis of mental disorder can in almost all cases be established by a pre-admission assessment. This should always be carried out by the admitting service, though it is good practice to obtain an independent medical certification before completing a compulsory admission order and in many jurisdictions this is a legal requirement. In the absence of a mental disorder, there may still be a need for an assessment of security need, but this may be better carried out by professionals other than the mental health team e.g. using the Level of Service Inventory-Revised (LSI-R, Andrews & Bonta 1995).

The purpose of the triage security items is to structure the decision making process when deciding what the appropriate level of therapeutic security might be for a person who is in need of admission to hospital from the criminal justice system – court or prison, or who has been referred for transfer to a more secure hospital or unit from a community mental health service. The DUNDRUM-1 triage items therefore are not intended to be used as a guide to the risk of future violence – the HCR-20 and other structured professional judgment and actuarial tools have already been validated for that purpose. Nor are the DUNDRUM-1 triage items intended to produce an actuarial score relating to fixed admission thresholds. These items should be regarded as a means of structuring the decision making process in accordance with factors that are relevant, in a way that is transparent and will lead to greater consistency. They may facilitate benchmarking between services and jurisdictions.

In general, a person who is mostly rated ‘4’ on these Triage Security Items is likely to require conditions of high therapeutic security at least for the early part of an admission to hospital; a person who is mostly rated ‘3’ is likely to need conditions of medium security, at least initially; a person who is mostly rated ‘2’ will benefit from treatment in conditions of psychiatric intensive care (acute low security), whether for a short or longer period; a person mostly rated ‘1’ should be safely treated and cared for in an open in-patient setting; a person mostly rated ‘0’ may be cared for in a community setting, including home treatment, crisis houses, high support community residences and other options. A person rated ‘0’ could also be followed by a prison in-reach mental health team. This does not preclude admission to hospital including secure placements, and / or the use of mental health legislation where appropriate. Further definitions of the various levels of therapeutic security have been defined elsewhere (Kennedy 2002)

Under the legal structures of some jurisdictions, courts have the power to determine that a person shall be admitted to a forensic mental health unit. This is often grounded in legislation creating a special status for selected secure hospitals, variously described as Special Hospitals (England & Wales), the State Hospital (Scotland), a designated centre (Ireland, Ontario) and other legal variants. The DUNDRUM-1 is designed as a structured professional judgment tool to assist the clinicians who act as expert witnesses or who are required to fulfil statutory obligations in advising the courts regarding the appropriateness of committal to a secure psychiatric facility. The Triage Security items may also be used as an audit tool for the appropriateness of such placement recommendations and orders.

The DUNDRUM-2 Triage Urgency items (described separately) are intended to provide a structure for deciding who on a waiting list for admission to a given level of security is the most urgent. In general, a higher score indicates the more urgent need. However at the time of drafting this first version, it is not clear that the DUNDRUM-2 Urgency items are logically or ethically simply additive. As clinicians, the authors are strongly of the opinion that clinical urgency should always take precedence over other non-clinical factors. In practice, there may be times when a legal obligation over-rules a clinical priority. This may have adverse health consequences for the more clinically urgent case. It is the responsibility of the clinicians and clinical managers to ensure that the legal decision maker is fully aware of the consequences of such exercise of legal power.

As for all structured professional judgment tools, the decision makers are not bound by the ‘result’ of the assessments. One highly rated item may be enough to require admission to the highest levels of therapeutic security given an individual context. Other factors that are not included in this toolkit may become relevant in an individual case.

**Triage Security Item 1: Seriousness of Recent Violence**

The seriousness or gravity of a risk is an aspect of dangerousness that is often missed by risk assessment tools. This item should be distinguished from the later item dealing with public confidence issues. Scott, in an influential early paper on risk assessment, defined dangerousness as a product of probability (risk) and the gravity of the risk in question. A person may be at high probability of some minor act, or at a low probability of some very serious act, such as homicide. Assessing the gravity of violence risk is therefore a legitimate element in the rational triage of those requiring psychiatric treatment. Eastman & Bellamy (1998) identified the seriousness of violent acts as the first element of a structured professional judgment manual for auditing security needs. Coid & Kahtan (2000) using a classification of seriousness of the most recent offence showed that this was one of the elements of an algorithm correctly describing the allocation of patients to various levels of therapeutic security.

The scientific evidence for specialisation in offending careers is easily overshadowed by evidence that offending is usually diverse. Evidence of specialisation is strongest for sexual offences (Stander et al 1989, Grubin et al 2001). Tracy et al (1990) found that the average seriousness of offences increased and specialization also increased as offenders became older and with each successive offence. Offenders released from prison in the USA were 53 times more likely than the general population to be rearrested for homicide over the next three years, while those released from prison whose most recent offence was homicide were 1.4 times more likely than other offenders to be rearrested for homicide, and many times more likely than the general population. Similar specialization emerged for all violent offences, rape, other sexual assaults, robbery, property offences and fraud (Langan & Levin 2002). Similar ‘specialization’ can be shown for mentally disordered arsonists (Rice & Harris 1996) and stalkers amongst others. See also Walker & McCabe (1973, vol 2 p194). Specialization and escalation are real phenomena, comparable to suicide research regarding ‘preferred method’ (Appleby et al 2001) and ‘lethality’ (Beautrais 2001).

Where there is a recent history of life-threatening violence, higher levels of therapeutic security will be required. This is not however the only determinant of the level of therapeutic security required, and other factors, as listed in this guide, should always be considered also. The seriousness of the risk of suicide is recognised as an important determinant of risk of suicide (see for example the S-RAMM), but in the context of this instrument we take seriousness as a guide to the level of therapeutic security required.

It follows that these two items are rated as ‘historical’. They should rely on behaviour for which there is at least prima facie evidence – charges pending, charges brought, facts proven on the balance of probabilities (civil standard), facts proven beyond reasonable doubt (criminal standard, e.g. facts proven but unfit to stand trial) convictions in court (beyond reasonable doubt). Assaults in hospital for which no charges were brought should be documented according to the date and time of contemporaneous description in the hospital notes.

NB All previous violence must be rated, even if the person was not mentally disordered at the time of past violence. Rate on the most serious violent act known.

NB If there is no current mental disorder (broadly defined), the correct rating is zero (0), because the person is not in need of psychiatric admission or follow up.

**Coding: TS1. Seriousness of Violence**

| 0 | No previous violence, or no current mental disorder (mental disorder includes adjustment reaction) |
| --- | --- |
| 1 | minimal degrees of violence, minimal threat to life. |
| 2 | Repetitive assaults causing injury such as bruising, that cannot be prevented by two-to-one nursing in open conditions. Less serious sexual assaults, (summary offence) |
| 3 | Use of weapons to injure, arson endangering life, assaults causing concussion or fractures to long bones, stalking with threats to kill. Single serious sexual assault, (indictable) |
| 4 | Homicide, stabbing penetrates body cavity, fractures skull, strangulation, serial serious (e.g. penetrative, indictable) sexual assaults, kidnap, torture, poisoning |

Information Quality: 0=no information; 1=staff observation only; 2=interview and staff observation; 3=family informants; 4=medical or police records.

**Triage Security Item 2: Seriousness of Self-Harm**

NB the previous item TS1 needs little adaptation to be applied to attempted suicide and self-harm. The aim here is to emphasise the seriousness of the attempt, with added weight given to the current suicidal intent. Although these factors can be found in risk assessment instruments for suicide, we are concerned here to assess the seriousness or gravity of the harm. For a fuller account of the risk of suicide and self harm see the S-RAMM, a structured professional judgment instrument (Bouch & Marshall 2003, Ijaz et al 2009, Fagan et al 2009)..

NB If there is no current mental disorder (broadly defined), the correct rating is zero (0), because the person is not in need of psychiatric admission or follow up.

**Coding: TS2: Seriousness of Self-Harm**

| 0 | No previous self-harm, or no current mental disorder (mental disorder includes adjustment reaction) |
| --- | --- |
| 1 | Self harm of minimal severity and minimal threat to life |
| 2 | Repetitive self-harm causing non-life-threatening injury that cannot be prevented by two-to-one nursing in open conditions |
| 3 | Use of potentially lethal means such as ligatures, arson, jumping to injure self, with continued suicidal intent |
| 4 | Near miss attempts at suicide, with continued suicidal intent – hanging with loss of consciousness, overdoses requiring ventilation or organ support, jumping from significant heights or arson requiring prolonged hospital treatment |

Information Quality: 0=no information; 1=staff observation only; 2=interview and staff observation; 3=family informants; 4=medical or police records.

NB All previous self-harm must be rated, even if the person was not mentally disordered at the time of past self-harm. Rate on the most serious self-harming act known.

NB If there is no current mental disorder (broadly defined), the correct rating is zero (0), because the person is not in need of psychiatric admission or follow up.

**Triage Security Item 3: Immediacy of Risk of Violence due to Mental Disorder**

The immediacy of a risk determines the extent to which high, medium or low levels of supervision are currently required. In higher levels of therapeutic security, higher staff-to-patient ratios ensure closer monitoring and greater opportunities for early de-escalation of any threat of violence. “Serious violence” here refers to violence rated ‘3’ or ‘4’ on item TS1 ‘seriousness of violence’.

There are various ways in which a risk may be immediate – an unassessed risk due to a mental disorder is for practical purposes unpredictable, and should therefore be regarded as immediate. Those with pervasive anger and resentment often have heightened sensitivity and may be explosive or provoked in response to minimal or mistakenly perceived ‘provocations’. Paranoid psychoses, acute schizophrenia or manic states may all be associated with such angry, sensitive mental states. A person who has a mental disorder co-morbid with intoxication or unmanaged withdrawal is likely to be labile in mood and similarly impulsive and unpredictable.

Scales such as the Dynamic Appraisal of Situational Aggression In Patient Version IV (DASA Ogloff & Daffern 2006) can be used to reliably rate the warning signs for immediate or short term risk of violence.

An acute relapse of a mental illness leading to such problems may be time limited. Such episodes may resolve with treatment in three to six months and may be managed in lower secure settings designed for short term care. Others may be anticipated to remain at risk for longer periods and may therefore require treatment in settings intended to cope with longer term continuing risk.

**Coding: TS3. Immediacy of Risk of Violence due to Mental Disorder**

NB If there is no current mental disorder (broadly defined), the correct rating is zero (0), because the person is not in need of psychiatric admission or follow up.

| 0 | No abnormality of mental state and /or no violence. (mental state includes current adjustment reactions) |
| --- | --- |
| 1 | Partially recovered from mental state that led to less serious violence or non-violent offence |
| 2 | Still in mental state that led to less serious violence |
| 3 | Partially recovered from mental state that led to serious violence |
| 4 | Still in the mental state that led to serious violence. |

Information Quality: 0=no information; 1=staff observation only; 2=interview and staff observation; 3=family informants; 4=medical or police records.

**Triage Security Item 4: Immediacy of Risk of Suicide**

Like the previous item TS3, this is a dimension which may influence the initial triage decision but should not be regarded as enduring – the rating can be revised down or up. See also the S-RAMM (Bouch & Marshall 2003, Ijaz et al 2009, Fagan et al 2009).

**Coding: TS4. Immediacy of Risk of Suicide**

NB If there is no current mental disorder (broadly defined), the correct rating is zero (0), because the person is not in need of psychiatric admission or follow up.

| 0 | No current abnormality of mental state (mental state includes symptoms of adjustment reaction) and /or no history of suicidal or self harming behaviour |
| --- | --- |
| 1 | Partially recovered from mental state that led to less serious self harm |
| 2 | Still in mental state that led to less serious self harm |
| 3 | Partially recovered from mental state that led to serious self harm (high lethality) |
| 4 | Still in the mental state that led to serious self harm (high lethality) |

Information Quality: 0=no information; 1=staff observation only; 2=interview and staff observation; 3=family informants; 4=medical or police records.

**Triage Security Item 5: Specialist Forensic Need**

There are persons for whom the recorded seriousness of violence and imminence of risk are not enough to fully describe the need for specialist forensic care and treatment.

When a person has a previous history of treatment in conditions of high or medium security, it may be presumed that on relapse they will need to return to the highest levels of security they have previously been allocated to. This has limited if any validity, and should be subjected to a structured reassessment of the current need as described by the totality of this guide.

One of the practical indicators of the level of therapeutic security currently needed is that the person has demonstrably exceeded the safe capacity of a well-organised therapeutically secure service at a lower level. Where there is any doubt, it is better to err on the side of caution if readmitting, and in the first instance readmit to a lower level of therapeutic security than before.

There are problems for which treatment can only continue in a therapeutically safe and secure environment. These are usually problems for which the therapist might be at risk in the course of treatment. Patients who incorporate clinicians into their delusional systems, patients in whom sadistic or expressively violent patterns of behaviour are prominent, arsonists or others may require a high level of therapeutic security for treatment to proceed. For practical purposes, specialist treatment programmes for such problems can often only be delivered in conditions of therapeutic security, at least initially.

**Coding: TS5. Specialist Forensic Need**

NB If there is no current mental disorder (broadly defined), the correct rating is zero (0), because the person is not in need of psychiatric admission or follow up.

| 0 | No history of mental disorder (mental disorder includes current adjustment reaction), **or** Co-operates with voluntary treatment, integrates into community mental health services, consents to all interventions recommended |
| --- | --- |
| 1 | Cannot cooperate with voluntary treatment, compliant when detained |
| 2 | Current mental state associated with violence, may include crisis or recall of former medium / high security patient |
| 3 | Arson, jealousy, resentful stalking, or exceeds capacity of PICU / low secure unit |
| 4 | Sadistic, paraphilias associated with violence, or exceeds capacity of medium security |

Information Quality: 0=no information; 1=staff observation only; 2=interview and staff observation; 3=family informants; 4=medical or police records.

**Triage Security Item 6: Absconding/Eloping**

One of the uses of therapeutic security is to prevent absconding (referred to in North American literature as ‘eloping’). Clinical risk management indications for preventing absconding include preventing suicide or self harm, and preventing harm to others. Learmont (1995) provides an algorithm for identifying those in need of increasing levels of security to prevent escape from within a secure setting. One of the factors identified by Learmont is ‘trust’.

This item should be rated conservatively – those who can safely be cared for at home or in an open setting with close nursing observations e.g. to prevent self harm or suicide, should not be moved to more secure settings.

Legal obligations may be imposed over clinical considerations at times, e.g. to ensure that those facing long sentences or currently serving long sentences do not abscond.

**Coding: TS6. Absconding/Eloping**

NB If there is no current mental disorder (broadly defined), the correct rating is zero (0), because the person is not in need of psychiatric admission or follow up.

| 0 | No history of mental disorder (mental disorder includes current adjustment reaction), **OR** Will not break off contact with mental health team in the community or prison in-reach mental health service |
| --- | --- |
| 1 | If absconded or broke off contact, would not present an immediate or grave danger to the public or to specific victims |
| 2 | Current risk of impulsive (opportunistic) absconding/escaping only, which could be prevented by admission to PICU |
| 3 | Currently pre-sentence and facing a serious charge **or** currently serving a long sentence, or capable of planning and deception in order to abscond/escape |
| 4 | Currently has not demonstrated capacity for trust in relation to absconding **and** Past history of absconding from custody at medium or high security levels; capable of planning, deception, corruption or coercion in order to abscond/escape; may be helped to abscond/escape by third parties |

Information Quality: 0=no information; 1=staff observation only; 2=interview and staff observation; 3=family informants; 4=medical or police records.

**Triage Security Item 7: Preventing Access**

There may be reasons why it is necessary to protect the person concerned from specific stressors e.g. the ready availability of drugs or intoxicants if these might otherwise be readily available, to prevent access to weapons, or to protect specific individuals or categories of person. This may include the ability to monitor and under certain defined circumstances to block communications e.g. in relation to the victims of stalking or threats, to other vulnerable or potential victims and access to pornography, violent material or other threatening material.

**Coding: TS7. Preventing Access**

NB If there is no current mental disorder (broadly defined), the correct rating is zero (0), because the person is not in need of psychiatric admission or follow up.

| 0 | No history of mental disorder (mental disorder includes current adjustment reaction) **OR** Can be trusted not to misuse intoxicants, weapons, communications, media or access to vulnerable persons without the need for imposed restrictions and monitoring in the community |
| --- | --- |
| 1 | Will comply with all aspects of risk management regarding restricted and monitored access to intoxicants, weapons, communications, media and access to vulnerable persons or potential victims while in hospital |
| 2 | Requires some restriction and monitoring of access to intoxicants, weapons, communications, media and access to vulnerable persons. Is sufficiently limited in PICU / acute low security due to impulsive, unplanned nature of actions |
| 3 | Ditto for medium security – will misuse if access is possible, is capable of some planning or deception to gain access to contraband or forbidden media / communications  **OR** needs to be separated from others he might have feuds / grudges against or who might have grudges against him |
| 4 | Ditto for high security – and has the capacity to obtain contraband, media, communications etc by means of planning, deception, corruption, coercion or the help of third parties,  **OR** needs protection from well-organised gangs/third parties |

Information Quality: 0=no information; 1=staff observation only; 2=interview and staff observation; 3=family informants; 4=medical or police records.

**Triage Security Item 8: Victim Sensitivity / Public Confidence Issues**

An awareness of the risks to others is an important part of the triage decision. Risks to others include those who have been the victims of explicit threats to kill or persistent unwanted attention (stalking). High-risk relationships may be relevant here, even when the third party wishes to have or resume full contact (battered spouses, children or parents).

Stranger victims or neighbours may object to the return of the person to their vicinity because of their fears or subjective discomfort.

Social and community considerations may also be relevant - local notoriety, media interest and the risk of revenge or reprisals against the person may all be relevant.

**Coding: TS8. Victim Sensitivity / Public Confidence Issues**

NB If there is no current mental disorder (broadly defined), the correct rating is zero (0), because the person is not in need of psychiatric admission or follow up.

| 0 | No history of mental disorder (mental disorder includes current adjustment reaction)  **OR** No local victim sensitivities or community sensitivities **AND** no high risk relationships |
| --- | --- |
| 1 | No long term local sensitivity or notoriety |
| 2 | Short-term or enduring family sensitivities or victim sensitivities |
| 3 | Significant local notoriety or local media interest.  **OR** Predictable potential victims (including vulnerable family members or high risk relationships) |
| 4 | Has national / media notoriety,  **OR** has made explicit credible threats to kill, to named individuals |

Information Quality: 0=no information; 1=staff observation only; 2=interview and staff observation; 3=family informants; 4=medical or police records.

**Triage Security Item 9: Complex Needs Regarding Risk of Violence**

This item can best be described as a qualitative ‘profile’ of the factors relevant to risk of violence, in so far as this relates to the level of therapeutic security required for safety and specialist treatment programmes to alleviate the combination of problems. As outlined in the introduction, this tool is intended to assist decision making regarding the level of security required..

The rating chosen here offers the opportunity to use the ‘Historical’ items of risk assessment instruments such as the HCR-20 as they were intended, as a guide to structured professional judgment. The ratings described below offer ‘profiles’ based on the most widely used static, background or historical risk factors to rate increasing complexity of treatment needs and need for therapeutic security.

NB This pattern needs little adaptation to describe risk of suicide (see for example S-RAMM). However a risk of suicide in the absence of a significant risk of violence is always manageable in open hospital or low-secure settings. Medium or higher levels of therapeutic security are required for prison to hospital transfers only when other factors intervene such as absconding risk (TS6) or institutional behaviour (TS10).

**Coding: TS9. Complex Needs Regarding Risk of Violence**

NB If there is no current mental disorder (broadly defined), the correct rating is zero (0), because the person is not in need of psychiatric admission or follow up.

| 0 | No history of major mental illness (as in HCR-20 H6). Other factors may be present, but this profile is best managed within the criminal justice system – see LSI-R or similar |
| --- | --- |
| 1 | NB No history of violence. Major mental illness (as in HCR-20 H6) is the only definite background/static risk item identified, may have co-morbidity (substance misuse, personality disorder) |
| 2 | Previous violence (as defined in HCR-20 H1) and current / recent violence in the context of major mental illness. Co-morbid problems if present are minor / not prominent |
| 3 | Previous serious violence in the context of major mental illness **and** substantial co-morbidity (complex problems) – i.e. major mental illness with one of the following: severe substance misuse problems (e.g. daily misuse or weekly binges), severe personality disorder (persistent even when mental illness and substance misuse are in remission) or other relevant significant historical/background risk factors (e.g. intellectual disability, acquired brain injury) |
| 4 | Current and / or previous serious violence **not** confined to the context of active symptoms of major mental illness;  **OR** co-morbid high score on the PCL-R or PCL-SV (threshold as in HCR-20 H7) |

Information Quality: 0=no information; 1=staff observation only; 2=interview and staff observation; 3=family informants; 4=medical or police records.

**Triage Security Item 10: Institutional Behaviour**

Berecochea and Gibbs (1991) found that behaviour during previous periods in custody was one of the classification factors relevant to the appropriate level of security for individuals, at least in prison. The behaviours rated here may also be relevant to moves between levels of therapeutic security.

**Coding: TS10. Institutional Behaviour**

NB If there is no current mental disorder (broadly defined), the correct rating is zero (0), because the person is not in need of psychiatric admission or follow up.

| 0 | No history of mental disorder (mental disorder includes current adjustment reaction),  **OR** None of the problem behaviours listed below for a proportionate period of time, with evidence of change |
| --- | --- |
| 1 | Socially embarrassing, undignified, disruptive, challenging or threatening behaviour when in the community; that might lead to arrest for public order or minor / non-violent offences or further damage to patient’s social network. But no habitual pattern of violence in hospital |
| 2 | Impulsive fire setting or other high risk behaviour in the community which can be managed in hospital with observation and behavioural programme. Bullying or coercive behaviour towards vulnerable fellow patients. Threatening to staff e.g. while incorporating into delusions. May have a pattern of previous violence while in hospital |
| 3 | Fire setting in hospital; barricading (without hostages) or roof-top protests in hospital or other secure settings as follower or without accomplices;. sexually active with vulnerable fellow patients (non-coercive); high risk threats of serious violence to staff and in-mates or patients; May have a history of previous serious violence while in hospital |
| 4 | Hostage taking in hospital or other secure institution; co-ordination of disturbances in hospital or other institution (i.e. a prime mover in such behaviour); necessity to separate from other specific persons to prevent harm to others (e.g. feuds). Fashioning weapons or other contraband within the secure setting;. sexually predatory/coercive behaviour towards vulnerable fellow-patients or in-mates |

Information Quality: 0=no information; 1=staff observation only; 2=interview and staff observation; 3=family informants; 4=medical or police records.

**Triage Security Item 11: Legal Process**

Note that the least restrictive option possible and acceptable to all should be preferred as the rating here. ‘All parties’ implies that the court should be satisfied with the proposed arrangement since the court is likely to have a veto.

**Coding: TS11. Legal Process**

NB If there is no current mental disorder (broadly defined), the correct rating is zero (0), because the person is not in need of psychiatric admission or follow up.

| 0 | No history of mental disorder (mental disorder includes current adjustment reaction) **OR** Community placement (out patient) legally possible and acceptable to all parties |
| --- | --- |
| 1 | Admission to local approved centre (e.g. open admission ward) legally possible and acceptable to all parties |
| 2 | Admission to low secure unit (e.g. PICU) legally possible and acceptable to all parties |
| 3 | Only admission to a forensic secure centre is acceptable to all parties. |
| 4 | Only admission to a forensic secure centre is legally possible |

Information Quality: 0=no information; 1=staff observation only; 2=interview and staff observation; 3=family informants; 4=medical or police records.
